# Rapid quantification of murine bile acids using liquid chromatography-tandem mass spectrometry

**DOI:** 10.1007/s00216-024-05668-0

**Published:** 2024-12-02

**Authors:** Sven Hermeling, Johannes Plagge, Sabrina Krautbauer, Josef Ecker, Ralph Burkhardt, Gerhard Liebisch

**Affiliations:** 1 ZIEL Institute for Food & Health, Research Group Lipid Metabolism, Technical University Munich, Munich, Germany; 2 Institute of Clinical Chemistry and Laboratory Medicine, University Hospital Regensburg, 93053 Regensburg, Germany

**Keywords:** Lipidomics, Bile acid, Plasma, Liver, Bile, LC–MS/MS

## Abstract

Interest in bile acids (BAs) is growing due to their emerging role as signaling molecules and their association with various diseases such as colon cancer and metabolic syndrome. Analyzing BAs requires chromatographic separation of isomers, often with long run times, which hinders BA analysis in large studies. Here, we present a high-throughput method based on liquid chromatography-tandem mass spectrometry to quantify BAs in mouse samples. After acidic protein precipitation in the presence of a comprehensive mixture of stable isotope-labeled internal standards (SIL-ISs), BAs are separated on a biphenyl column by gradient elution at basic pH. Quantification is performed using a six-point calibration curve. Except for the separation of β- and ω-muricholic acid (MCA) species, a rapid separation of 27 BA species was achieved in a run time of 6.5 min. Plasma quality controls (QCs) were used to evaluate intra- and inter-day precision. The CV was less than 10% for most BA species and exceeded 20% only for glycohyodeoxycholic (GHDCA) and taurohyodeoxycholic acid (THDCA) due to the lack of a corresponding SIL-IS. The limit of quantification (LoQ) was tested using diluted QCs and was found to be compromised for some BA species as a result of insufficient isotopic purity of the SIL-IS, leading to significant interference with the respective analyte. Finally, we tested the mouse sample material requirements for plasma, bile, and liver samples and determined BA concentrations in C57/BL6N wild-type mice. In conclusion, the LC–MS/MS method presented here permits a rapid and reproducible quantification of the major murine BAs.

**Supplementary Information:**

The online version contains supplementary material available at 10.1007/s00216-024-05668-0.

## Introduction

In recent decades, interest in bile acid (BA) research has increased as they have been recognized as signaling molecules that influence various systemic processes [1, 2]. Alterations in BA profiles have also been associated with several diseases, including colorectal cancer, microbial dysbiosis, and metabolic syndrome [3–7]. These findings shifted the perception of BAs from simple lipid solubilizers to complex signaling molecules [2]. Moreover, strong interactions between the gut microbiota and BA metabolism have been shown to influence health and disease [8–11].

BAs that are synthesized from cholesterol in the liver through a series of cytochrome P450-catalyzed hydroxylations, cleavage of the C25-C27 side chain, and conjugation to glycine and taurine are referred to as primary BA [12]. After synthesis, BAs are stored in the gallbladder as bile. Upon secretion after food intake to facilitate lipid digestion, BAs are subjected to deconjugation, epimerization, and de- and re-hydroxylation by bacterial enzymes from the gut microbiota, resulting in secondary BA such as ursodeoxycholic acid (UDCA), lithocholic acid (LCA), and deoxycholic acid (DCA). BAs are taken up by enterocytes via passive and active absorption and transported back to the liver via the portal vein referred to as entero-hepatic circulation [1]. In contrast to humans, where glyco-conjugated BA are present at high concentrations, taurine-conjugated BAs dominate in mice. In addition, mice possess CYP2C70, a cytochrome p450 enzyme, which converts the primary BA chenodeoxycholic acid (CDCA) to α-, β-, and ω-muricholic acids (MCAs). Notwithstanding these differences between humans and mice, mice are widely used as a model system to study the role of BAs and their metabolism in disease research [13].

A major challenge for the quantification of BAs from either organism is the separation of isomeric species differing only in the position and orientation of hydroxy groups. Reversed-phase LC coupled to tandem mass spectrometry (LC–MS/MS) is the method of choice for the analysis of BA species due to its high specificity and sensitivity [14, 15]. Numerous methods for the quantification of BAs have been published, mostly to study human BA profiles that do not cover MCAs [16–18]. Methods that measure a wide range of BAs, including MCAs, typically have long run times of more than 20 min [19–27], which limits sample throughput. Few methods report BA analysis, including MCAs, with run times less than 20 min [28, 29]. However, short run times may compromise the separation of isomeric MCA species, such as the co-elution of TαMCA and TβMCA [28]. Another shortcoming of existing methods is that stable isotope-labeled internal standards (SIL-IS) for MCA species are often not included as a prerequisite for accurate quantification [30].

Here, we report a rapid and sensitive LC–MS/MS method for the quantification of 27 BA species in mice with a total run time of 6.5 min, using a set of 22 isotopically labelled standards to ensure high data quality. The method was evaluated for mouse plasma, bile, and liver samples.

## Materials and methods

### Chemicals and reagents

Ammonium acetate, ammonia solution (25%), and acetonitrile were purchased from Merck (Darmstadt, Germany). LC–MS grade methanol and hydrochloric acid were purchased from VWR Int. GmBH (Darmstadt, Germany). Ultrapure water was obtained from a Milli-Q EQ 7000 system (Merck, Darmstadt, Germany).

BA standards, both stable isotope labeled internal standards (SIL-IS), and unlabeled analytes (see list of abbreviations) were purchased from the following manufacturers: GHDCA, GUDCA, GLCA from Steraloids (Newport, USA); D4-TγMCA, TγMCA, D4-GCDCA, D4-GCA, TβMCA, TαMCA from Cayman Chemicals (Ann Arbor, USA); D5-γMCA, D5-βMCA, D5-αMCA, γMCA from IsoSciences (Ambler, USA); D4-GLCA, D4-GUDCA, D4-GDCA from CDN Isotopes (Pointe-Claire, Canada), D4-UDCA from Larodan (Solna, Sweden); D5-TCA, D5-TUDCA, D5-TCDCA, D5-TDCA, D5-TLCA, D4-TβMCA, D4-TαMCA, GβMCA, βMCA, αMCA, D5-HDCA from Toronto Research Chemicals (Toronto, Canada); and remaining BAs from Merck (Darmstadt, Germany).

Calibrator stock solutions were prepared by the addition of the unlabeled analytes to methanol, followed by stepwise dilution to the respective concentrations. Quality control (QC) stocks were based on pooled human plasma, in part supplemented with BA species. Supplementation was performed dropwise from methanolic BA standard solutions while stirring the plasma. Stirring was continued in the cold for at least a further 60 min, and QCs were stored in aliquots at − 80 °C. IS stock solution contained D4- and D5-labeled BAs were prepared in methanol. All solutions were stored at − 20 °C.

### Murine samples

Plasma, liver, and bile samples were obtained from wild-type C57/BL6N mice fed ad libitum a chow diet and bred at the specific pathogen free facility of the ZIEL Institute for Food & Health, Technical University of Munich. Breeding was performed in accordance to the relevant ethical guidelines (German Animal Welfare Act) under controlled conditions (group-housing, 55% relative humidity, 23 °C ambient temperature, 12-h/12-h light–dark cycle). Euthanization was performed using CO_2_ asphixiation and cardial puncture at 15–16 weeks of age. Samples were shock frozen in liquid nitrogen and stored at − 80 °C for up to 6 months.

Prior to BA extraction, liver tissue was homogenized in isopropanol (0.05 mg wet weight/µL) using 1.4 mm ceramic beads and a FastPrep 24 tissue homogenisor (Bertin Technologies SAS, Mantigny le Bretoneux, France) set to 6 m/s for 2 × 30 s. Bile obtained by gall bladder puncture was diluted 1:1000 in ultrapure water. Blood samples were promptly transferred into EDTA tubes and centrifuged at 4 °C and 1500 × g for 10 min to obtain plasma.

### Sample perparation

Sample preparation was based on a previously published method of acidic protein precipitation with minor adjustments [18]. Sample processing was performed with a sample volume of 50 µl, corresponding to 0.05 µL of undiluted bile and 1 mg of liver tissue homogenisate (10 µl of liver homogenate + 40 µl water), respectively. Unless stated otherwise, 50 µL of plasma was used. Calibrator samples were prepared by dilution of 5 µL stock solution in 45 µL ultrapure water. Plasma QC samples were subjected to sample processing without further dilution. Each batch contained IS and solvent blanks. All samples were spiked with 10 µL of IS-stock solution, excluding solvent blanks. For precipitation of proteins, 15 µL 1 M hydrochloric acid and 500 µL acetonitrile were added to the samples, followed by thorough vortexing for 1 min. The resulting precipitate was centrifuged at 14,000 × g for 15 min, and the supernatant was transferred and evaporated using a vacuum centrifuge. Each sample was then resuspended in 100 µL of 30% (v/v) methanol in ultrapure water by vortexing for 1 min, followed by 10 min of ultrasonification. For removal of unsolved matter, the samples were then centrifuged for 15 min at 14,000 × g, and the supernatant was transferred to glass vials.

### LC–MS/MS analysis

Separation and detection of BAs was achieved by liquid chromatography-tandem mass spectrometry (LC–MS/MS). A PAL RSI 534 (CTC Analytics, Zwingen, Switzerland) was used in combination with an Agilent 1290 Infinity II HPLC system (Agilent, Waldbronn, Germany) for automated sample injection and analyte separation. For analyte detection, a QTRAP 6500^+^ triple quadrupole mass spectrometer (Applied Biosystems, Darmstadt, Germany) was used in conjunction with ESI in negative ion mode.

A sample volume of 3 µL was injected and separated on a Kinetex Core–Shell Biphenyl column 50 × 2.1 mm with a particle size of 2.6 µm (Phenomenex, Aschaffenburg, Germany) kept at 50 °C, using gradient elution at a constant flow rate of 600 µL/minute. The mobile phases were 100% ultrapure water (A) and methanol (B), both containing 0.01% NH_3_ and 10 mM ammonium acetate. The linear gradient starts at 10% B, an increase to 47% B at 0.1 min, 49% at 1.2 min, 58% at 2.3 min, 68% at 4.7 min, and 100% at 4.8 min before returning to 10% at 5.8 min for re-equilibration of the column until 6.5 min. The MS was used in negative ion mode with the following settings: 400 °C ion source heater temperature, 50/70 psi source gas 1/2 and 40 psi curtain gas, and − 4500 V ion spray voltage. Analyte monitoring was performed using scheduled multiple reaction monitoring (sMRM) with a target scan time of 0.4 s and unit resolution. The list of mass transitions is shown in Table S1.

### Analyte quantification

Analyte peak areas were normalized to the peak area of their corresponding IS as indicated in Table S1. Quantification of BAs was based on a six-point calibration curve of the respective peak area ratios. Calibration curves were calculated by linear regression without weighing. The resulting slope was used for the calculation of analyte concentrations. Since GγMCA, GαMCA are not commercially available and thus not added to the calibrator mix, and the slopes of TγMCA and TαMCA were used instead, respectively. Interferences introduced from insufficient isotopic purity of stable isotope-labelled IS species were corrected by background subtraction. This was based on experimentally determined analyte-to-IS peak area ratios determined from a set of *n* = 5 IS blanks.

Peak integration was performed using the MQ4 integration algorithm in Sciex OS 3.1 (Applied Biosystems, Darmstadt, Germany). The exported data was further processed in self-programmed Excel macros, performing the analysis including calculation of analyte-to-IS ratios, correction of IS interference, calculation of regressions, and calculation of the analyte concentrations from response factors.

### Method validation

Accuracy and reproducibility of the method were evaluated by using serum QCs used in patient diagnostics. These QCs were supplemented with MCA species. Reproducibility in mouse samples was evaluated in pooled plasma and bile samples, as well as in biological replicates of liver samples from BL6/N mice, respectively. Carryover was evaluated as follows: the highest calibrator level was injected five times, followed by the injection of three solvent blanks. The carry-over was calculated as the analyte area measured in the respective blanks relative to the calibrator, expressed in percentage.

## Results and discussion

The aim of the present study was to extend our previously established method for the quantification of human serum BAs in routine laboratory diagnostics [18, 30], to rodent BA profiles applicable to different sample materials. Furthermore, we aimed to keep the method run time short in order to achieve sufficient sample throughput and to increase the sensitivity by using a state-of-the-art LC–MS/MS system.

### Fragmentation and separation of BA

As a first step, commercially available BAs were used to find and optimize mass spectrometric settings in negative ion mode including declustering potential (DP), collision energy (CE), and collision cell exit potential (CXP). As previously described [18], the main fragment ions of *m/z* 74 and 80 were observed for glycine and taurine conjugated species, respectively. Most unconjugated BAs did not show a prominent product ion. Therefore, we used a mass transition without fragmentation, and only for UDCA, CA and DCA additional fragment ions were included to increase confidence in their identification and quantification (Table S1).

**Fig. 1:**
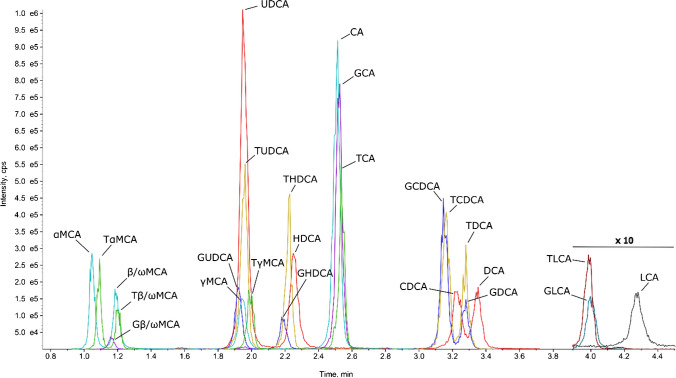
Chromatogram of a representative BA calibrator sample. Isomeric BAs measured with the same mass transition are shown in the same color

Since isomeric BAs cannot be differentiated by mass spectrometry, they need to be separated by chromatography. Previously, we successfully used a water–methanol gradient and an RP18 column at basic pH to separate human BA [18]. Despite adaptation and optimization of the LC-gradient, we were not able to efficiently separate MCA species (data not shown). Therefore, we tested a biphenyl column for MCA species separation. While this stationary phase showed superior resolution of MCA species, co-elution of conjugated and unconjugated species of the same BA required a decrease in ammonia concentration from 0.1 to 0.01% to achieve co-elution of conjugated and unconjugated species of the same BA (data not shown). Co-elution of free BAs and its glyco- and tauro-conjugates not only facilitates easy identification in the absence of the corresponding BA standard, but may also be advantageous to account for the absence of a corresponding SIL-IS (see discussion below). Using these parameters, we were able to separate 24 of the 30 targeted BA species in less than 6.5 min (Fig. 1). It was not possible to separate isomeric β- and ω-MCA, as well as their conjugates, differing only in the orientation of the hydroxy group at C6 position.

Compared to most existing methods for the separation of murine BA species [19–28], this method allows a more than twofold increase in sample throughput. Sangaraju et al. [29] report a run time of 10 min, also using basic LC mobile phases. However, they did not report on ω-MCA species, which may co-elute with β-MCA similar to the present method. Furthermore, they chose to report α-MCA and β-MCA (including their tauro-conjugates) together, as these isomers were not separated by the baseline.

### Quantification of BA

Quantification was based on 22 stable isotopically labeled (SIL) BA species used as IS and 6-point calibration lines for most of the target analytes. As previously demonstrated, suitable ISs (best matching stable isotope labelled) are essential to compensate for matrix effects and to achieve accurate and reproducible BA quantification [30]. Therefore, for those species for which no matching SIL-IS was available, the IS with the closest retention time was used for quantification (Table S1). Calibration lines were linear in the validated range with Pearson coefficients > 0.99 (Table 1). Of note, the calibrator was made from a BA standard mixture prepared for routine diagnostics of human serum supplemented with MCA species. No commercially available standard was available for GαMCA and GγMCA. Therefore, calibration lines for TαMCA and TγMCA were used. As the analytical response of tauro- and glycol-conjugates may not be similar, these concentrations should be considered as an approximation. Compared to existing methods [19–29], however, the comprehensive set of SIL-BAs included here represents an advance, as matching SIL-ISs were missing for only 6 analytes.

**Table 1:** Method performance for individual BA species

Analyte	LoQ [nM] *	Tested upper calibration limit [µM]	Mean/target concentration [µM]		Accuracy [%]		Precision [%]			
					QC1	QC2	Intra-day		Day-to-day	
			QC1	QC2			QC1	QC2	QC1	QC2
**αMCA**	78	1.5	0.1	0.47	-	-	2.5	1.8	8.6	5.8
**TαMCA**	71	4.5	0.63	1.89	-	-	6.7	6.4	4.2	7.5
**βωMCA**	78	1.5	0.22	0.44	-	-	6.1	3.4	19.3	6.4
**GβωMCA**	78	1.5	1.04	0.47	-	-	-	-	-	-
**TβωMCA**	71	4.5	0.75	2.04	-	-	6.3	8.5	10.5	14.6
**γMCA**	8	1.5	0.12	0.52	-	-	4.6	4.6	6.7	1.7
**TγMCA**	71	4.5	0.69	2.07	-	-	4.8	7.5	2.9	5.2
**UDCA**	18	7.7	1.81	5.53	87	97	1.9	0.9	5.8	7.1
**UDCA-MS2**	181				87	100	6.0	3.7	4.0	8.3
**GUDCA**	47	5.5	4.73	6.97	125	127	3.1	1.4	6.9	5.2
**TUDCA**	11	13.4	1.13	7.64	93	95	4.3	10.6	14.3	12.6
**HDCA**	24	3.4	0.24	1.9	-	-	3.9	2.7	4.5	11.4
**GHDCA**	15	1.9	0.15	1.21	-	-	7.0	4.2	29.7	33.5
**THDCA**	3	6.9	0.27	2.45	-	-	12.1	7.7	23.5	19.1
**CA**	63	6.3	0.63	3.87	107	109	4.6	4.6	2.5	2.9
**CA-MS2**	6				97	95	6.5	1.8	7.9	5.1
**GCA**	33	29.5	3.33	18.49	96	96	10.5	2.7	7.0	4.6
**TCA**	152	12.2	1.52	8.05	80	90	5.4	8.5	11.9	2.6
**CDCA**	70	2.7	0.7	2.02	97	106	1.5	2.2	3.5	1.1
**GCDCA**	37	16.1	3.7	11.39	105	105	1.7	1.0	3.9	5.7
**TCDCA**	25	14.9	2.51	10.76	83	86	5.9	3.4	5.4	7.1
**DCA**	52	2.2	0.52	1.6	97	101	2.3	3.8	5.1	7.8
**DCA-MS2**	5				96	100	1.8	3.8	5.7	1.8
**GDCA**	76	6.6	0.76	4.11	105	105	2.7	3.0	8.9	7.6
**TDCA**	5	7.0	0.52	4.04	102	104	9.2	5.7	10.6	8.1
**LCA**	8	1.0	0.08	0.4	111	113	2.5	0.8	2.5	4.3
**GLCA**	18	1.6	0.18	1.05	94	102	2.5	4.2	8.8	4.3
**TLCA**	12	1.7	0.12	0.97	117	116	9.4	11.2	4.6	11.7

Displayed are limits of quantification (LoQ; *derived from serial tenfold dilutions of QC1), upper calibration limit tested (highest concentrated calibrator), accuracy (when target concentrations were available) and intra- and day-to-day precision for serum quality control samples QC1 and QC2 (*n* = 4 replicates). For UDCA, CA, and DCA, the data refer to the MS parameter listed in Table S1 either without or with (MS2) fragmentation

### Validation of the method

The current method is intended for research purposes, not for patient diagnosis. Therefore, we decided to focus method validation on key analytical metrics, rather than following the comprehensive guidelines typically used for biomedical assays (see for example [31]). The performance of our method was first evaluated by assessing intra- and inter-day precision and accuracy for two serum quality control (QC) samples prepared from pooled human serum. Precisions for almost all BAs and both QCs were better than 10% CV (Table 1), demonstrating good reproducibility of the method. Higher variation was observed for QC1 for β- and ω-MCA due to concentrations close to the limit of quantification (LoQ; see below) and for inter-day CVs of GHDCA and THDCA for both QCs. No matching SIL-ISs were available for GHDCA and THDCA, which most likely caused the high analytical variation. Due to the lack of appropriate SIL-ISs, target concentrations were not available for HDCA and its conjugates (D5-HDCA has recently become commercially available). Since target values were also not available for MCAs added to the QCs, we did not calculate accuracies for these analytes. The accuracies for the remaining BA species were within ± 20% of the target values established for the human BA species. Only GUDCA showed a systematic shift in accuracy, most likely due to concentration deviation in the calibrator, as the reproducibility was excellent and both QCs showed similar variation.

The next step was to determine the limit of detection (LoD). Unfortunately, for the majority of the analytes, the IS blanks interfered with the SIL-IS due to insufficient isotopic purity (data not shown). For these analytes, the application of S/N to estimate the LoD is impossible. Therefore, we decided to roughly estimate the limit of quantitation (LoQ) from a serial dilution of QC1 (1:10, 1:100, 1:1000, and 1:10000; *n* = 4, respectively). The concentration levels for which both the CV was < 20% and the accuracy of the dilution was within ± 20% were defined as LoQ. For most BA species, the LoQs were less than 100 nM and ranged from 3 to 152 nM. Due to the tenfold dilution steps, the LoQs shown in Table 1 should be considered as estimates. It should also be noted that analyte interference resulting from SIL-IS can increase the LoQ. For example, TCA showed the highest LoQ and D4-TCA showed a fraction of 0.36% unlabeled analyte. Therefore, the use of isotopically pure SIL-ISs could significantly improve the sensitivity of the analysis in the low nM range.

For free BAs, the MS2 transitions for CA and DCA showed lower LoQs. Furthermore, quantification using MS2 transitions should be considered more specific than without fragmentation, and therefore, MS2 transitions should be preferred for quantification. However, for UDCA, fragmentation leads to a significant decrease in sensitivity, and therefore, quantification may only be possible without fragmentation.

Next, sample carryover was evaluated by repeated injection of the highest calibrator, followed by solvent blanks. Although carryover was less than < 0.2% for most BA species, a blank is recommended after samples with very high concentrations to avoid misquantification.

### Method application to mouse plasma, bile, and liver samples

**Fig. 2:**
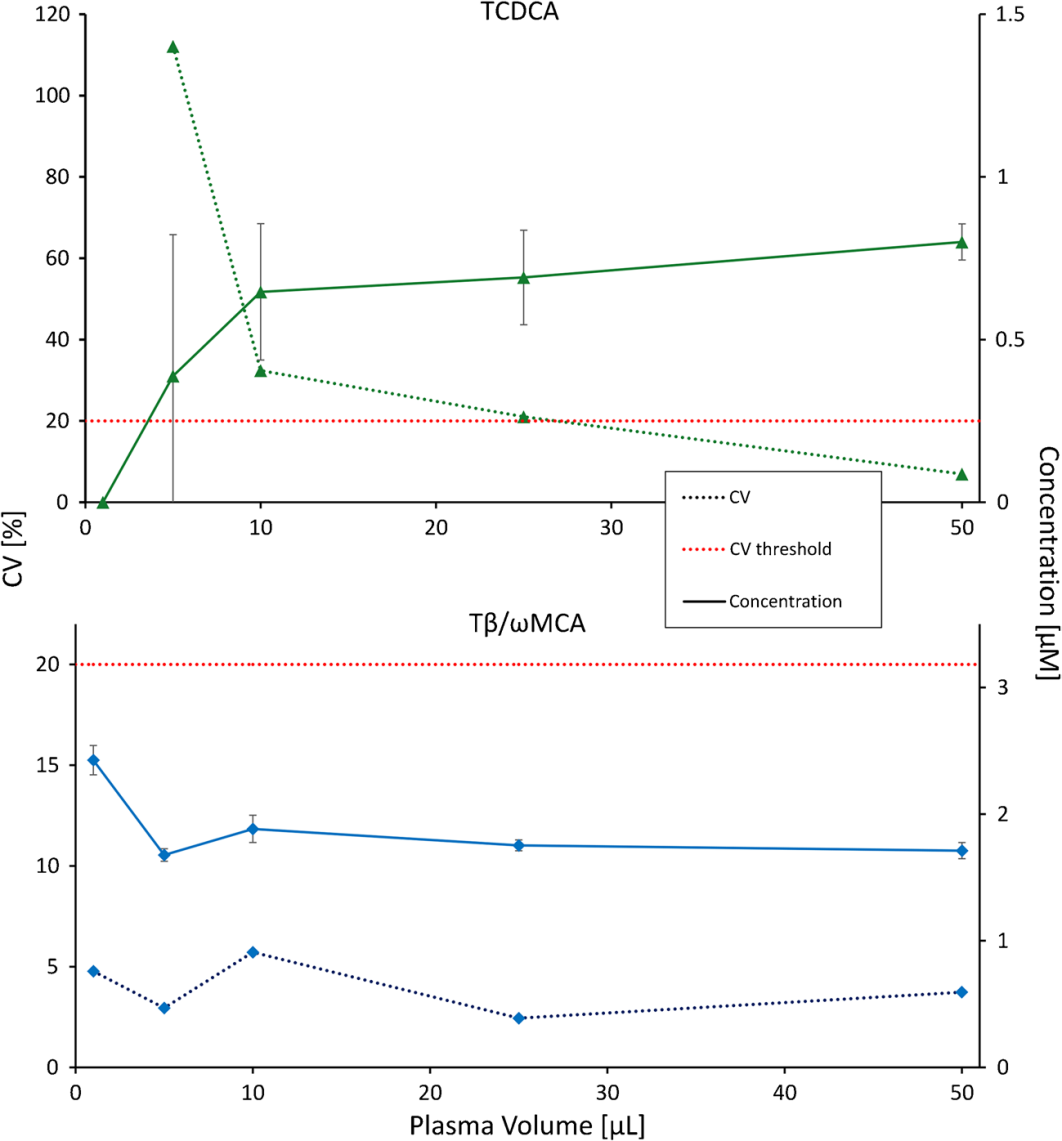
Accuracy and repeatability as a function of the plasma volume used. Precisions (dashed) and concentrations ± SD (solid) of TCDCA (green, upper panel) and TβωMCA (blue, lower panel) for *n* = 4 replicates of a plasma sample as a function of the volume used for protein precipitation. The CV threshold for accurate quantification is highlighted by a dashed red horizontal line

In addition to spiked human serum QCs, the performance of the method was tested in mouse plasma, bile, and liver samples. For plasma, sample volume requirements were tested by using 1, 5, 10, 25, and 50 µL as sample volume and filling each to a total volume of 50 µL with water (*n* = 4, respectively). The CV and precision of the dilution (based on 50 µL sample volume) were calculated. While the reproducibility was still sufficient for several BA species at 1 µL plasma volume, the dilution integrity was not met, also due to insufficient isotopic purity of SIL-IS (see above). As expected, CVs increase with lower volumes, as shown in Fig. 2 for TβωMCA and TCDCA. Using the same criteria as for the determination of the LoQ (see above), the following analytes could be quantified at 1 µL (βωMCA), 5 µL (TUDCA, TCA, CA, and TβωMCA), 10 µL (TDCA, DCA, TαMCA), 25 µL (UDCA, TCDCA), and 50 µL (HDCA, THDCA, TγMCA). For the quantification of major BA, 5 µL can be considered as sufficient sample amount, which represents a very economical use of sample material. However, for the quantification of minor BA, as shown in Fig. 2, larger volumes of up to 50 µL plasma are required. To cover the major BA species in bile, 50 µL of a 1000-fold diluted sample (equivalent to 0.05 µL of native bile) and 1 mg of liver tissue are required. BA retention times did not shift in these samples, permitting identification of BA species (see Figure S1 for representative chromatograms for mouse plasma, bile, and the liver).

**Fig. 3:**
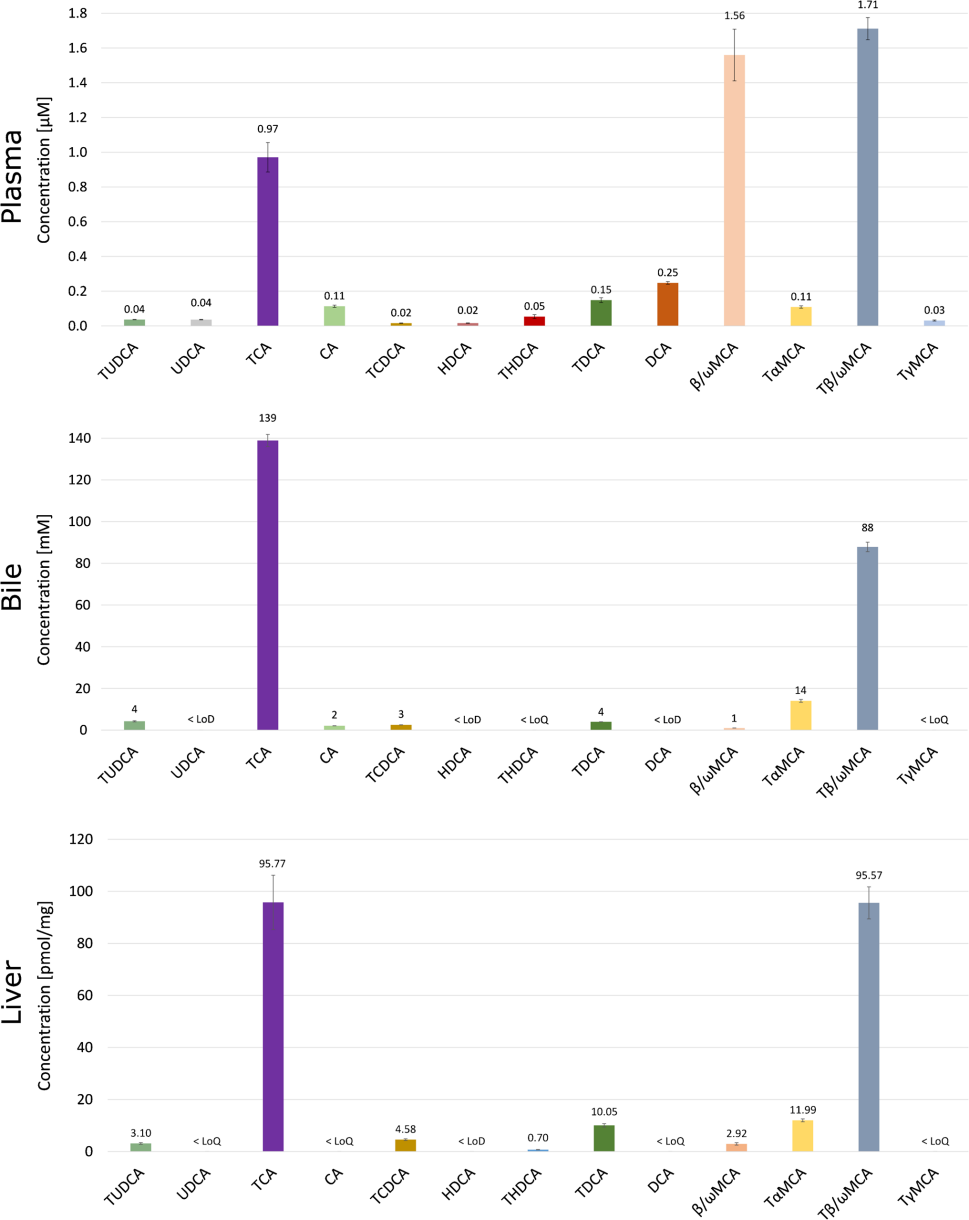
BA concentrations in murine plasma, bile, and liver. Bile acid concentrations in plasma, bile, and liver samples from WT mice (*n* = 3–4) are shown. Plasma sample volume was 50 µL. Bile and plasma data are based on pooled samples. Concentration is shown in µM, mM, and pmol/mg wet tissue ± SD for plasma, bile, and the liver, respectively. The mean concentration of the respective BA species is given above the bar

Finally, we determined BA concentrations in wild-type C57/BL6/N mice fed a chow diet. As expected, the plasma profiles differed from those in the liver and bile (Fig. 3). While secondary and unconjugated primary BA such as DCA, TDCA, CA, and βωMCA are detectable at relatively high levels in plasma, this is not the case for bile and the liver. As expected, bile and liver BA profiles are dominated by conjugated primary BA such as TCA and TβωMCA, and only traces of other BAs are detectable due to hepatic metabolism of these gut microbiota-derived metabolites [13, 32]. The observed concentrations were in good agreement with previously published concentrations and profiles of BAs in plasma, bile, and the liver [13, 33–35].

## Conclusion

We aimed to develop a fast, accurate, and versatile method for reproducible quantification of BAs from mouse samples using LC–MS/MS to meet the demand for high-throughput BA quantification driven by the increasing interest in their role in health and disease. Our method achieves baseline separation of most major BAs in 6.5 min. To the best of our knowledge, this is the fastest published method for murine BA quantification. For some BA species, insufficient isotopic purity of the SIL-ISs results in significant overlap with the corresponding analyte, compromising the LoQ and highlighting the need for isotopically pure SIL-ISs. In conclusion, this BA quantification method provides a valuable tool for reliable high-throughput quantification of BAs in research applications which was recently demonstrated also for analysis of cecal samples after bead beating [36].

## Supplementary Information

Below is the link to the electronic supplementary material.

Supplementary file1 (PDF 459 KB)

## Abbreviations

BA: Bile acid
CA: Cholic acid
CDCA: Chenodeoxycholic acid
DCA: Deoxycholic acid
HDCA: Hyodeoxycholic acid
LCA: Lithocholic acid
LoQ: Limit of quantification
MCA: Muricholic acid
prefix G: Glyco-conjugate
prefix T: Tauro-conjugate
QC: Quality control
SIL-IS: Stable isotope-labeled internal standard
UDCA: Ursodeoxycholic acid

## Declarations

### Statement on animal welfare

Breeding, handling, and sampling of mice were performed in accordance to a breeding permit under §11 of the German Law for Animal Welfare, and no animal experiments were performed as defined under §7 Sect. (2).
