# Supplementary material for: Rapid quantification of murine bile acids using liquid chromatography-tandem mass spectrometry
Source: Anal Bioanal Chem. 2024 Dec 2;417(4):687–96. doi: 10.1007/s00216-024-05668-0 (PMC11772536; doi:10.1007/s00216-024-05668-0)
Supplement: Supplementary file 1 — Supplementary file1 (PDF 459 KB) [file 216_2024_5668_MOESM1_ESM.pdf]

Supplementary Data

Rapid quantification of murine bile acids using liquid chromatography-tandem mass spectrometry

Sven Hermeling [1,2], Johannes Plagge [1], Sabrina Krautbauer [2], Josef Ecker [1,2], Ralph Burkhardt [2], Gerhard Liebisch [2,*]

1) ZIEL Institute for Food & Health, Research Group Lipid Metabolism, Technical University Munich, Germany

2) Institute of Clinical Chemistry and Laboratory Medicine, University Hospital Regensburg, Germany

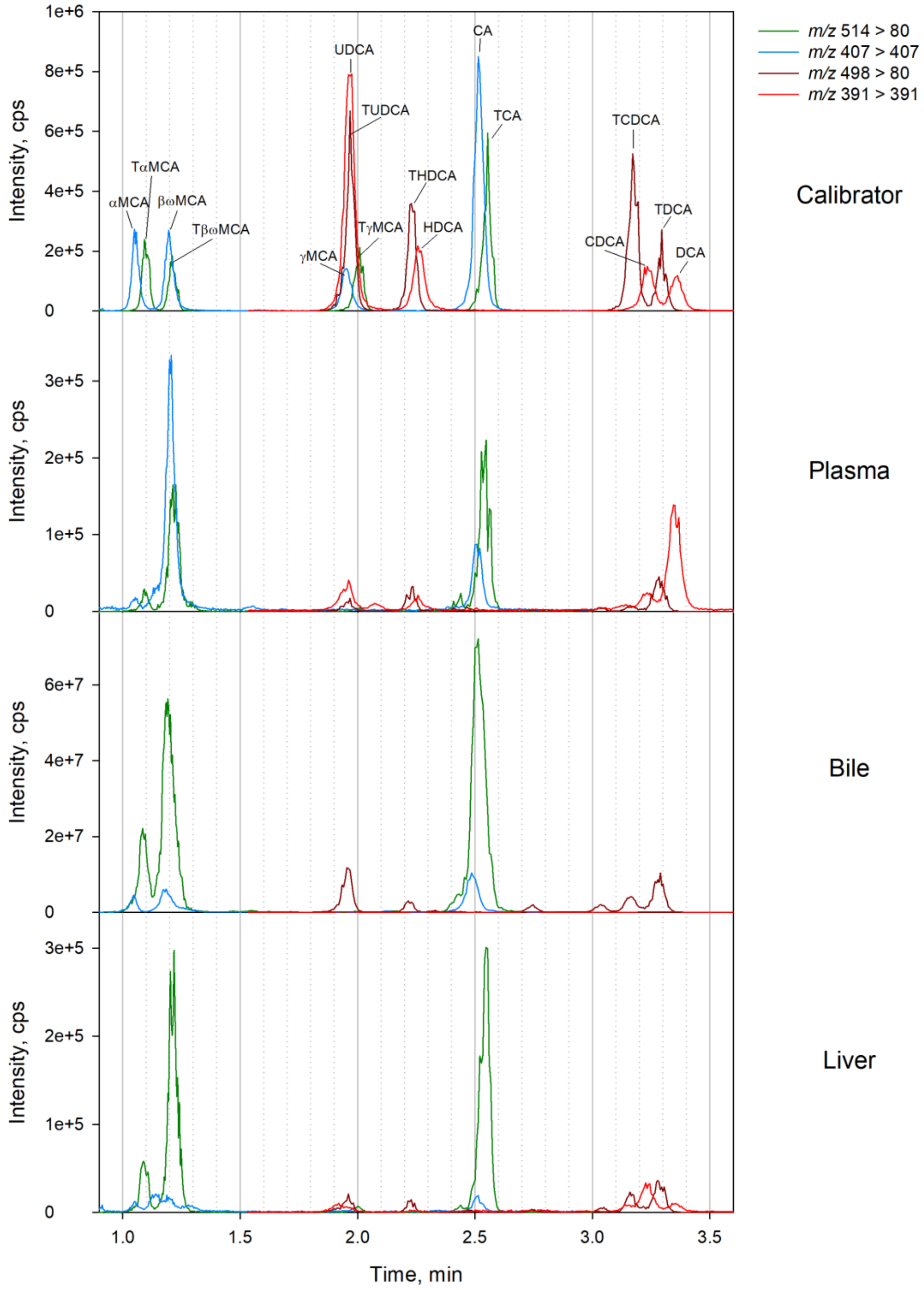

Figure S1: Chromatogram of calibrator, murine plasma, bile and liver.
Displayed are SRM traces for calibrator level 3, plasma (sample volume 50µl), bile and liver samples.

**Analyte**	RT [min]	Mass transition [*m/z*]	DP [V]	CE [V]	IS
**GαMCA**	1.06	464.3 → 74	-110	-94	D5-αMCA
**αMCA**	1.07	407.3 → 407.3	-125	-30	D5-αMCA
**TαMCA**	1.13	514.3 → 79.9	-240	-156	D4-TαMCA
**GβωMCA**	1.21	464.3 → 74	-110	-94	D5-βMCA
**βωMCA**	1.22	407.3 → 407.3	-125	-30	D5-βMCA
**TβωMCA**	1.25	514.3 → 79.9	-240	-156	D4-TβMCA
**GUDCA**	1.99	448.3 → 74	-85	-84	D4-GUDCA
**γMCA**	1.99	407.3 → 407.3	-125	-30	D5-γMCA
**GγMCA**	2.02	464.3 → 74	-110	-94	D5-γMCA
**TγMCA**	2.06	514.3 → 79.9	-240	-156	D4-TγMCA
**UDCA**	2.00	391.3 → 391.3<br>391.3 → 373.35	-155	-30<br>-45	D4-UDCA
**TUDCA**	2.02	498.3 → 79.9	-175	-164	D5-TUDCA
**GHDCA**	2.26	448.3 → 74	-85	-84	D5-HDCA
**THDCA**	2.28	498.3 → 79.9	-175	-164	D5-HDCA
**HDCA**	2.29	391.3 → 391.3	-155	-30	D5-HDCA
**CA**	2.55	407.3 → 407.3<br>407.3 → 343.2	-125	-30<br>-45	D4-CA
**GCA**	2.58	464.3 → 74	-110	-94	D4-GCA
**TCA**	2.60	514.3 → 79.9	-240	-156	D5-TCA
**GCDCA**	3.23	448.3 → 74	-85	-84	D4-GCDCA
**TCDCA**	3.23	498.3 → 79.9	-175	-164	D5-TCDCA
**CDCA**	3.28	391.3 → 391.3	-155	-30	D4-CDCA
**TDCA**	3.35	498.3 → 79.9	-175	-164	D5-TDCA
**GDCA**	3.36	448.3 → 74	-85	-84	D4-GDCA
**DCA**	3.40	391.3 → 391.3<br>391.3 → 345.2	-155	-30<br>-45	D4-DCA
**TLCA**	4.07	482.3 → 79.9	-165	-120	D5-TLCA
**GLCA**	4.09	432.3 → 74	-105	-74	D4-GLCA
**LCA**	4.32	375.3 → 375.3	-155	-30	D4-LCA
**D5-αMCA**	1.06	412.3 → 412.3	-125	-30	-
**D4-TαMCA**	1.13	518.3 → 79.9	-240	-156	-
**D5-βMCA**	1.21	412.3 → 412.3	-125	-30	-
**D4-TβMCA**	1.25	518.3 → 79.9	-240	-156	-
**D4-GUDCA**	1.98	452.3 → 74	-85	-84	-
**D5-γMCA**	1.98	412.3 → 412.3	-125	-30	-
**D4-UDCA**	1.99	395.3 → 395.3<br>395.3 → 377.3	-155	-30<br>-45	-
**D5-TUDCA**	2.01	395.3 → 377.4	-175	-164	-
**D4-TγMCA**	2.05	503.3 → 79.9	-240	-156	-
**D5-HDCA**	2.28	518.3 → 79.9	-155	-30	-
**D4-CA**	2.55	396.3 → 396.3<br>396.3 → 347.2	-125	-30<br>-45	-
**D4-GCA**	2.57	411.3 → 347.2	-110	-94	-
**D5-TCA**	2.59	411.3 → 411.3	-240	-156	-
**D4-GCDCA**	3.22	468.3 → 74	-85	-84	-

**D5-TCDCA**	3.22	519.3 → 79.9	-175	-164	-
**D4-CDCA**	3.26	452.3 → 74	-155	-30	-
**D4-GDCA**	3.34	503.3 → 79.9	-85	-84	-
**D5-TDCA**	3.34	395.3 → 395.3	-175	-164	-
**D4-DCA**	3.39	395.3 → 395.3	-155	-30	-
		395.3 → 349.2		-45	
**D5-TLCA**	4.05	452.3 → 74	-165	-120	-
**D4-GLCA**	4.08	395.3 → 349.2	-105	-74	-
**D4-LCA**	4.30	395.3 → 395.3	-155	-30	-

Table S1: BA analytical details.
Shown are retention time, mass transition, declustering potential (DP), collision energy (CE), and internal standard (IS) used for quantification for the monitored bile acid species. For the unconjugated BA UDCA, CA and DCA, two mass transitions were monitored.
